# Persistence of immunity to hepatitis B vaccine as infants, 17 years earlier

**DOI:** 10.22088/cjim.9.2.184

**Published:** 2018

**Authors:** Zohreh Azarkar, Azadeh Ebrahimzadeh, Gholamreza Sharifzadeh, Masood Ziaee, Mohammad Fereidouni, Fatemeh Taheri

**Affiliations:** 1 Infectious Diseases Research Center, Birjand University of Medical Sciences, Birjand, Iran.; 2 Asthma, Allergy and Immunology Research Center, Medical Sciences of University, Birjand, Iran.

**Keywords:** Hepatitis B, Vaccination, Immunity

## Abstract

**Background:**

In Iran since 1992, hepatitis B vaccination was a part of the national vaccination program. Hepatitis B vaccination is effective in the epidemiology of hepatitis B. The aim of this study was to evaluate the long – term persistence of immunity.

**Methods:**

This cross-sectional analytical study was conducted on children and adolescents aged between 6-18 years in Birjand, who received a three – dose hepatitis B vaccination in accordance with the national immunization program. No students were infected with hepatitis B. Antibody titer higher than10 IU/L was considered positive.

**Results:**

A total of 530 patients (307 boys and 223 girls) were recruited for the study of which 44% had positive antibody titer (≥10 IU / L). The geometric concentration mean (GMCs) of antibody in subjects was 64.9±34.2, HBS antibody titer was positive in 40.4% of the boys and 59.6% of the girls. A significant difference in antibody titers was observed in terms of gender and according to the time elapsed since the last vaccination. Antibody titer in children older than 13 years had passed since their last vaccination and was significantly less than those children younger than thirteen years old had passed since their vaccination logistic regression analysis showed that the only predictive factor of anti-HBS low titer (<10 IU/L) is elapsed time of vaccination.

**Conclusions:**

Based on results of this study, hepatitis B vaccine has created a good level of protection in 44% of the adolescents after 17 years.

Hepatitis B infection is a major health problem worldwide, more particularly in Asia. It can with different degrees (1). lead to serious complications such as cirrhosis and hepatocellular carcinoma (2). According to the World Health Organization (WHO) two million people are infected with hepatitis B virus worldwide more than 350 million people are chronically infected, and approximately two million people die of hepatitis B-related complications annually (3). The prevalence of hepatitis associated with HIV varies across the globe. Positive HBS antigen prevalence is less than 2% in the general population of the developed countries, such as Western Europe and North America. The rate is 2-8% in Asian countries and more than 8% in Africa and East Asia (4). In the city of Birjand in eastern Iran, incidence of HBV has been reported 1.3%, which is by far lower than the rest of the country and other Asian countries (5, 6). but chronic hepatitis B is the most common cause of hepatocellular carcinoma and liver failure (7). One of the main ways to control hepatitis B infection is the widespread use of hepatitis B vaccine. Since 1991, WHO offered a vaccination program against HBV in all countries, universal immunization program (6, 7). Hepatitis B vaccination has been part of the national immunization program in Iran from 1992. Initially, the vaccination program against hepatitis B for neonates was set for 0-1.5-9 months; however, it changed to 0-2-6 months from 2005 (8).

The immunity persistence of childhood vaccination of hepatitis B is one of the debatable issues. Immunity period of hepatitis B full vaccination depends on antibody titer created and its stable time length to a higher concentration of antibody titer of immunogenicity in the serum (7, 8). In a study in 2004 in Iran, antibody titer of children was examined 10 years after the infant’s vaccination. The results showed that 58% of children had protective antibody titer (10 IU/L) after ten years (3). In Iran, information about the long-term performance of the vaccine is limited and incomplete. Since hepatitis B vaccination is effective in its epidemiology, this study was conducted to evaluate its long term persistence of immunity.

## Methods

This cross-sectional analytical study was conducted from 2015 (September - December) on children residing in Birjand aged between 6 and 18 years who received the three-dose hepatitis B vaccine in accordance with the national immunization program. The sample was selected by multistage cluster sampling method. First, the city of Birjand was divided into four districts based on geographical situation. A list of school districts in each locality namely: elementary, middle and high schools was obtained from the Office of Education of Birjand. Two girls schools and two boys schools were selected randomly from the list of schools (in total, 24 girls’ and 24 boys’ schools). 

In each school in each grade, one class was selected randomly according to the population of each class, and several students from each class were systematically selected at random. A sample size of 534 people was estimated based on the formula. After selecting the sample, a written consent was obtained from parents for inclusion, and a self-made questionnaire was completed for the students based on the project objectives. 

The questionnaire, whose content validity was approved by five faculty members, consisted of information on demographics and vaccination status of specific diseases including hepatitis B. The vaccination basis was health vaccination cards of students in their school files. Inclusion criteria were all children who received three doses of HBV vaccine. Children who had received immunoglobulin, blood products, and other immunodeficiency drugs in the past three months or incomplete hepatitis B immunization were excluded. 

**Serologic Test: **After permission was obtained and coordination made with the Office of Education, 3 ml blood samples were taken and stored in serum isolation at -20 °C until the required tests were performed. After collecting samples, serum titer of hepatitis B surface antibody (anti-HBS) (bioELISA anti HBS Bio kit, Barcelona, Spain), hepatitis B surface antigen (HBS Ag) (Enzygnost HBS Ag5.o, Dade Behring Inc. Newark USA), hepatitis B core antibody (anti- HBC) (bio ELISA anti HBC, Bio kit, Barcelona, Spain) were conducted using ELISA method. At this stage, those with positive HBS Ag or anti-HBC were excluded (4 individuals were: HBS-Ag positive or HBS –Ab positive then). Finally, 530 patients were enrolled. All these people had received Engerix B vaccine made in Cuba or Iran with 10 microgram dose within 0-1.5-9 or 0-1-6 months. Antibody titer less than 10 Iu/L was considered negative and 10 IU/L was considered safe (according to the mentioned catalog kit).

**Statistical Analysis: **The data collected were analyzed in SPSS-16 software using Mann-Whitney, Kruskal-Wallis (given the non-normal distribution of antibody titer), chi-square, Pearson correlation coefficient, and logistic regression. The significant level was set at α=0.05.

## Results

This study included 530 children and adolescents aged between 8 and 18 years (307 males and 223 females) with the mean age of 13.1±2.3 years. All subjects were negative HBS Ag and negative anti-HBC. Demographic and epidemiological data of the subjects are displayed in (table 1). From these, 223 (44%) had positive antibody titer (≥10 IU/L). The geometric concentration mean (GMCs) of antibody in subjects was 64.9±34.2 (with a minimum of zero and a maximum of 250). The mean BMI in our study was 22.3±2.4 (with a minimum of 18.85 and a maximum of 37.17). In 40.4% of the boys and 59.6% of the girls, HBS antibody titer was positive. A significant difference in antibody titers was observed in terms of gender and elapsed time after vaccination.

Antibody titer was significantly less in children with over 13 years than those with less than 13 years from their last vaccination. While the mean antibody titer was higher in girls than boys, the difference was not statistically significant (table 2). A negative, significant correlation was found between antibody titer and age. Finally, to determine factors associated with antibody titer less than 10 IU/L, logistic regression analysis showed that the only predictive factor is elapsed time from the last vaccination (table 3).

**Table 1:** Characteristics of students stratified by anti-HBS titer

**HBS-Ab**	**>10** **N(%)**	**<10** **N(%)**	**Chi-square test**
**Gender**			
Male female	124(40.4) 109(48.9)	183(59.6) 114(51.1)	P=0.05
			
**Education**			
Illiterate Primary Secondary and Highschool graduate College	8(53.3) 42(53.2) 25(41.7) 45(44.1) 32(48.5)	7(46.7) 37(46.8) 35(58.3) 57(55.9) 34(51.5)	P=0.64
**Mother occupation**			
Housekeeper Staff Self-employed	129(48.3) 16(41) 7(46.7)	138(51.7) 23(59) 8 (53.3)	P=0.69
**Vaccine status**			
vaccinated≤13years vaccinated>13years	152(49.5) 84(36.7)	155(50.5) 145(63.3)	P=0.003

**Table 2:** Comparison of GMCs by gender and vaccine status in students

	**Frequency**	**Mean±SD**	**Mann-whitney test**
**Gender**			
Male Female	307 223	61.4±31.1 69.4 38.5	P=0.06
**Vaccine status**			
vaccinated≤13years vaccinated>13years	307 229	71.1±40.52 53.8±25.32	p<0.001

GMCs: Geometric Concentration Mean

**Table 3:** Logistic regression analysis of variable related to variation of anti-HBS<10 IU/L

**Parameter**	**Odds ratio**	**95% CI**	**P-value**
Gender: female vs. male	0.71	0.501-1.003	0.052
Vaccine status: vaccinated≤13years vaccinated>13years	1 1.69	1.19-2.4	0.023

95% CI: Confidence Interval

## Discussion

This seroepidemiologic retrospective study was performed to evaluate the immunity of children and adolescents who received vaccination during the neonatal period. According to the literature, this seems to be the first study to assess the immunity persistence in children and adolescents up to 17 years from the time of vaccination. Vaccination of Iranian infants with a dose of 10 micrograms of recombinant hepatitis B vaccine in accordance with the 0-1.5-9 months program of 1992 was included in the national immunization program. Since 1998, recombinant Engerix-B vaccine manufactured by SmithKline, Belgium has been imported and used. However, long after the recombinant Herbert Biovac-HP vaccine (made in Cuba) with the same dose was used. From 2005 to present, the Iranian recombinant hepatitis B vaccine with a dose of 10 micrograms is used in accordance with the 0-2-6 table of the national immunization program (7, 8).

 The criterion for immunity in vaccination with hepatitis B vaccine is concentration of anti-HBS antibody in the serum. If the concentration of the produced antibody is higher, the achieved immunity will be greater (9). Immunity duration achieved by a whole series of hepatitis B vaccination depends on antibody titer and stability duration of serum titer (10, 11). Based on this study, 56% of the subjects over 17 years after vaccination were reported to have lost antibodies of the vaccine. Moreover, the average concentration of antibodies underwent a significant reduction. If the findings of this study are compared with previous studies in Iran, it seems that similar results were obtained, and about half of the children lost the acquired immunity by HBV vaccination in less than 15 years (9, 11). In various studies carried out on diverse and high-risk populations years after HBV vaccination, 15-50% of the vaccinated children have lost measurable anti-HBS titer 5-15 years after vaccination, and average concentrations of antibody also reduced significantly (12, 13). 

In some investigations, 5 years after vaccination, children born from mothers who are chronic carriers of hepatitis B, 16% of immunized children lost the acquired immunity (3). In Seto and Shih’s study, 6-7 years after vaccination 43% and 81% of immunized children, had lost their immunity (12, 14). The results of all above studies suggested that antibody titers decreased over time. In this study, 44% of subjects had antibody titer more than 10 IU/L (which had been accepted as a protective titer) and 15% had antibody titer less than 10 IU/L (1-9 IU/L). It was found that the persistence of antibody titer exists after 13 years, which is consistent with the findings in some studis (15-17). There is scientific evidence that people whose antibody titer reduces to less than 10 IU / L or their antibody titer becomes negative, are still protected against hepatitis B, because the immunologic memory to HBS Ag continues (13, 18). Even in these people, there is a guarantee to experience a sharp increase in antibody titer in the exposure of hepatitis B virus (19-22). Furthermore, based on some studies, it has been proven that people with antibody titer less than 50 IU/L after hepatitis B vaccination, may have hepatitis B. Accordingly, in some European countries, the minimum protective titer of 100 IU/L is considered (16, 23). 

The duration of immunity persistence after hepatitis B vaccination and its role in immunity is still controversial. In fact, there is no scientific evidence that immunity is only based on the anti-HBS amount. Therefore, based on the WHO recommendation, people with antibody less than 10 IU/L are considered immunized due to the strong role of the response of cellular immunity against hepatitis B (16, 24). There were limitations in this study, because this was a retrospective seroepidemiology study. There was a lack of sufficient information in retrospective studies because these studies are usually inevitable. One of the important limitations of this study is the lack of information about the original antibody titer after vaccination in the neonatal period. So it is not clear that anyone who has antibody titre below 10 IU/L whether having primary and secondary decreased antibody titer happens over time.

Today, according to some studies, one of the reasons associated with lower anti-HBS titers is introduced as a routine immunization for infants, and they believed that vaccination during adolescence contributes significantly to hepatitis B vaccination associated with this progressive evolution of the immune system during childhood. Immune system in infants with poor performance of T cells, low interaction between T and B cells and immunoglobulin limitation cause reduced antibody response (25, 26). These observations cause strategy of hepatitis B vaccination time to be revised for a better and more effective response to the prescribed vaccine. In addition, another limitation of this design is the lack of information about the status of mothers in term of hepatitis B immunity or catching. The presence of anti-HBS in serum of some mothers may affect response to hepatitis B vaccine in newborns and reduce the immunogenicity of hepatitis B at birth (4). In spite of that, the, results of one study have shown that 22% of babies born of mothers infected with HBV do not have the ability to produce anti-HBS after three doses of hepatitis B vaccine, and in majority of these individuals HBV-DNA was observed in their peripheral blood mononuclear cells (PBMC). Although there is not HBV-DNA in PBMC of infants who were born of infected mothers, they had immunity antibody response to the vaccine (8, 27). One important factor that affects immune response is body mass index (BMI) in which unfortunately in this study, the calculated BMI is associated with the time of investigation of antibody titers during the study period.

In conclusion, Hepatitis B vaccine has a very good performance to prevent hepatitis B and has created a good level of protection in 44% of the adolescents after 17 years. It is suggested to conduct a study on virus – exposed youth to evaluate the effectiveness and immunity continuity of hepatitis B virus vaccination.
